# Listening to the patient as a possible route to cost-effective rehabilitation: a case report

**DOI:** 10.1186/1752-1947-6-19

**Published:** 2012-01-17

**Authors:** Attilia Grandi, Marcella Mazzola, Lucia Angelini, Matteo Chiappedi

**Affiliations:** 1 Don C Gnocchi Foundation, Piazzale Morandi 6, 20162 Milan, Italy

## Abstract

**Introduction:**

Adolescents with cerebral palsy often do not need a specific rehabilitative treatment; however, when specific needs are expressed, clinicians should listen and try to answer them.

**Case presentation:**

We present the case of a 17-year-old Italian male patient with hemiplegia who had received standard physiotherapy and, ultimately, after a period of adapted physical activity performed in a group, was under consideration for discharge. However, due to unsatisfactory hand control, he asked for help to reach a personal goal, the ability to drive a motorbike, without surgery. Functional taping showed efficacy, but was neither cost-effective nor practical for the patient and his family; by contrast, a dynamic orthosis associated with training in a real-life environment was instead successful.

**Conclusion:**

The present case underlines the importance of considering solutions involving the motivation and compliance of the patient in order to improve his activity and participation.

## Introduction

Adolescents with a mild form of cerebral palsy often do not need a specific rehabilitative treatment [1], however, they can present specific requests which need to be considered in order to increase their wellbeing. From a biopsychosocial point of view, as suggested by the World Health Organization [2], their needs should be understood not only in terms of impairment (problems in body functions and/or structures), but also with respect to activity limitations (difficulties in executing activities) and/or participation restrictions (limitations an individual may experience in social involvement) in a dynamic interaction with environmental factors. The recent version of the International Classification of Functioning, Disability and Health (ICF) for Children and Youth [3] adds that the temporal perspective should not be neglected, with the related changes in terms of body functions and structures, but also of interests, desires and relevant activities and participations.

The reported case highlights the importance of paying attention to specific needs indicated by a patient, but also the utility and cost-effectiveness of using orthosis in this context.

## Case presentation

Our Italian male patient was first seen in the Rehabilitation Center of the Don Gnocchi Foundation when he was 15. He was a monochorionic twin born preterm through a Cesarean performed under urgency conditions due to placental abruption during the 33rd week. He had right hemiplegia; brain magnetic resonance imaging showed a large area of brain pores in the left middle cerebral artery territory, with indirect signs of a previous thrombosis of the same vessel. He was treated in a Rehabilitation Center up to the age of 12, with physiotherapy focused on his lower limbs and gait. When admitted to our Center, he participated in a group receiving adapted physical activity, because physiotherapy was not judged to still be appropriate; at the age of 17, he was considered for discharge.

By that time he was able to walk independently, with a high level of recruitment of his right plantar flexors; his left pelvis and shoulder were elevated as a functional compensation. His right upper limb was flexed at the elbow with flexion and ulnar deviation of his wrist further increased on action. Bimanual activity was possible, but conditioned by a synkinetic pattern of his right upper limb towards his left one. His cognitive functioning was normal.

Before discharge, he asked for help to improve his control of the right elbow flexion, which was particularly disturbing during fast movements. He considered this improvement to be an important task because he wanted to ride a motorbike but he was unable to control it during abrupt decelerations or when the road was bumpy.

His level of autonomy, as evaluated by the Pediatric Evaluation of Disability Inventory, and his results in the Gross Motor Function Measure and in the Melbourne Upper Limb Function are shown in detail in Table 1. Observing the videotape of these tests, it was clear that his main problems were the control of elbow flexion and forearm supination.

**Table 1:** Results for our patient in different tests.

	Initial evaluation	With functional taping	With Lycra orthosis
**Gross Motor Function Measure **(%)	94	96	96
**Melbourne Upper Limb Function **(%)	81.15	90.16	90
**Pediatric Evaluation of Disability Inventory **(%)	100	100	100

Both our patient and his parents refused surgery and drug administration; neither orthopedic surgery nor botulinum toxin injection were therefore considered as options. As a first attempt, we proposed a functional taping meant to control the elbow flexion and increase his thumb extension and forearm supination. With this taping, his performance improved (see Table 1). As revealed by the videotape, the improvement was mainly related to the increased control of the elbow, even in situations of fast movement. Our patient declared that he was no longer disturbed by the synergic flexion of his right upper limb while walking, that bimanual tasks were easier and 'more natural' to perform and that he could make better use of proprioceptive inputs.

**Figure 1:**
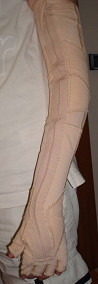
**Flexa dynamic orthosis**. This version of Flexa is shaped like a long glove, going from the elbow to the metacarpal joints. Ties can be regulated in order to obtain forearm supination and elbow extension.

However, he was not still happy with this solution since every third day he had to be brought to our Center by his parents to change the taping and thus his desire to be more independent was not achieved. For this reason we proposed to use Flexa, a Lycra orthosis shaped like a long glove (from the elbow to the metacarpal joints) with ties to grant forearm supination and elbow extension (see Figure 1).

With this treatment, despite the fact that his performances were almost unchanged (see Table 1), he was able to start driving the motorbike in his backyard, later following his parents' car in a country road and a couple of months later on his own.

## Discussion

Several aspects of interest and consideration arise from this case report. The first consideration is that the well known quote 'doctor knows best' needs to be rethought because the role of the patient, with his or her unique needs and desires, must be included in any decision regarding his or her health. A further limitation in Italy is the imperativeness of parental consent to any medical treatment. Although, in theory, it is possible to challenge parents' decisions in the courts, this option is usually exploited only in the most severe and life-threatening situations. Prioritizing patients in therapeutic decisions has many positive consequences. First, it can lead to the choice of a potentially suboptimal therapeutic strategy (in our case, using an orthosis without orthopedic surgery or botulinum toxin injection) in the interests of improving the patient's well-being, sense of independence and compliance with the treatment. Second, rehabilitative goals should be decided in agreement with both the patient and their family, preventing treatment refusal [4]. Third, dealing with patients who are growing, treatment may be tailored to their changing interests and desires: a child may be interested in playing, an adolescent may want to drive a motorbike to allow him to share experiences with other adolescents and as a step toward independence.

A second point of interest is the role of orthosis in the rehabilitation of adolescents. Flexa, the Lycra dynamic orthosis we chose, has a non negligible cost, but due to the relevant benefit reported even with the static variant of a similar orthosis [5], we hypothesized that it could prove useful as well [6]. This kind of orthosis is often used in rehabilitative practice as an adjuvant to improve the efficacy of botulinum toxin injection and/or orthopedic surgery; to the best of our knowledge, however, no published studies have assessed its real efficacy. We believe that this case report shows that the orthosis is effective even if we had to use it on its own, given the lack of consent to other more invasive procedures.

Moreover, when compared in terms of mere costs with functional taping over a one-year period, the orthosis proves to be profitable (see Table 2). Additionally, in this analysis the 'total cost' we calculated does not include costs we could not quantify, such as the time spent by the parents to bring our patient to the Rehabilitation Center (considering a total of about one and a half hours lost per taping, this could lead to 150 hours per year), the increased feeling of dependence from both parents and therapist (a factor which is always dangerous in adolescence) and the poorer quality of life of both the patient and his family. These factors, however, notably increase the real cost of the taping treatment.

**Table 2:** Comparison of costs between functional taping and Flexa (a Lycra orthosis).

	Functional taping	Flexa
**Material^a ^**(euros)	70	535
**Therapist^b ^**(euros)	1250	50
**Total **(euros)	1320	585

^a^Cost for functional taping based on an average of 0.70 euros per taping; cost for the Lycra orthosis taken from the catalog of the manufacturer ('Progettiamo autonomia'); ^b^cost is calculated assuming a full cost of 50 euros/hour for 0.5 hours of work per taping. The orthosis is supposed to last 1 year; in the same time span, the patient should need approximately 100 tapings.

A third consideration lies in the importance of using tests, comparing pre-and post-treatment results, as tools for guiding rehabilitation. Our patient had a slight change in the Gross Motor Function Measure, but the improvement in the Melbourne Upper Limb Function was significant not only in terms of raw scores but also as an improvement of his functioning [2,3]. Used in this way, tests provide a quantitative base for treatment evaluation without misleading the clinical judgment [7].

## Conclusion

We believe that the biopsychosocial framework portrayed by the World Health Organization in the ICF classifications [2,3] could be useful to better understand and to plan the best possible solution for rehabilitative needs.

These should include the use of orthoses, which can be as cost-effective as any other kind of assistive technology, even in resource-limited settings [8].

## Consent

Written informed consent was obtained from the patient's parents (his legal guardians) for publication of this case report and any accompanying images. A copy of the written consent is available for review by the Editor-in-Chief of this journal.

## Competing interests

The authors received a research grant from 'Progettiamo autonomia' (Barbieri SRL).

## Authors' contributions

AG performed the physiatric and general medical exam of our patient. MM performed the functional taping and all functional evaluation of our patient. All authors contributed to writing the manuscript, which they all read and approved in its final version.
